# Comparative Analysis of Single-Stage vs. Multiple-Stage Interventions in the Management of Subarachnoid Hemorrhage in Patients with Multiple Intracranial Aneurysms

**DOI:** 10.3390/jcm14134705

**Published:** 2025-07-03

**Authors:** Oday Atallah, Khadeja Alrefaie, Amr Badary

**Affiliations:** 1 Department of Neurosurgery, Evangelic Hospital Oldenburg, Carl Von Ossietzky University Oldenburg, 26122 Oldenburg, Germany; 2 Kuwait Institute for Medical Specialization, Sulibekhat 15503, Kuwait; 19200612@rcsi.com; 3 Department of Neurosurgery, Medical University Lausitz, 03048 Cottbus, Germany; amr.badary@hotmail.com

**Keywords:** subarachnoid hemorrhage, multiple intracranial aneurysms, single-stage intervention

## Abstract

**Background/Objectives:** Subarachnoid hemorrhage (SAH) due to ruptured intracranial aneurysms remains a critical neurosurgical emergency with high morbidity and mortality. The presence of multiple intracranial aneurysms (MIAs) in SAH patients presents a therapeutic challenge, particularly in choosing between single-stage and multiple-stage interventions. In patients with aneurysmal SAH and multiple intracranial aneurysms, we compared single-stage vs. multiple-stage interventions regarding vasospasm occurrence, complication rates, and short-term neurological outcomes in a retrospective cohort. **Methods:** This retrospective cohort study included 44 patients diagnosed with aneurysmal SAH and at least one additional unruptured aneurysm. Patients were categorized based on the intervention strategy. A “single-stage” intervention was defined as treatment of both the ruptured and all unruptured aneurysms in the same operative session. A “multiple-stage” intervention referred to a planned approach in which additional aneurysms were treated in separate, subsequent procedures. Clinical severity was assessed using scores. Aneurysm characteristics and treatment modalities were recorded. Outcomes were analyzed and compared between intervention groups. Statistical analysis was performed, with *p* < 0.05 considered significant. **Results:** The cohort included 44 patients with a total of 109 aneurysms. Most patients were female (68.2%), with a mean age of 54.5 years. The majority of aneurysms were small- to medium-sized and commonly located in the anterior circulation. Among the patients, 19.0% underwent single-stage interventions, and 28.6% underwent multiple-stage procedures. Vasospasm occurred significantly more often in the single-stage group (83.9% vs. 46.2%, *p* = 0.028). No significant difference was found in hospital stay duration between groups. The MRS scores showed a trend toward worse outcomes in the single-stage group (*p* = 0.060), as did the rates of post-operative neurological deficits (*p* = 0.079). **Conclusions:** In patients with SAH and MIAs, single-stage interventions may increase vasospasm risk. Although they offer logistical benefits, outcomes should be interpreted with caution given baseline differences and limited statistical adjustment.

## 1. Introduction

Subarachnoid hemorrhage (SAH) is a life-threatening neurological emergency, most commonly caused by the rupture of cerebral aneurysms [1]. Although it accounts for only about 5% of all stroke cases, SAH is associated with disproportionately high morbidity and mortality rates, making it a critical focus in neurovascular care [2].

The underlying pathophysiology involves complex interactions among vascular anomalies, hemodynamic stressors, and biological mechanisms that promote aneurysm formation and rupture [3,4,5]. Known risk factors—such as hypertension, smoking, and genetic predisposition—contribute to both aneurysm development and the likelihood of rupture, informing current preventive and screening strategies [6].

Early diagnosis through neuroimaging modalities such as computed tomography (CT) and magnetic resonance imaging (MRI) is essential, followed by prompt therapeutic intervention to secure the aneurysm and prevent rebleeding [7,8]. The clinical severity of SAH is typically assessed using grading systems such as Hunt and Hess, Fisher, and World Federation of Neurological Surgeons (WFNS) scores, which help predict outcomes and guide treatment decisions [9,10].

Despite advances in diagnostic and therapeutic approaches—including microsurgical clipping and endovascular coiling—the optimal management strategy for patients presenting with multiple intracranial aneurysms (MIAs) remains a subject of ongoing debate [11,12,13,14,15,16,17,18]. In particular, the decision between single-stage vs. multiple-stage interventions may significantly impact both procedural risks and long-term outcomes.

This study aims to evaluate the influence of surgical strategy—single-stage vs. multiple-stage intervention—on clinical outcomes in patients with SAH due to MIAs. Additionally, we analyze demographic and clinical characteristics, aneurysm distribution and treatment modalities, and the implications of each management approach in routine clinical practice.

## 2. Methods

This retrospective cohort study involved 44 patients diagnosed with SAH and concurrent unruptured cerebral aneurysms. This was a retrospective, single-center observational cohort study conducted and approved by the relevant institutional review board. Data extraction was conducted by two independent reviewers using a standardized case report form. Variables collected included demographic data; risk factors; aneurysm characteristics; imaging findings; surgical details; clinical severity scores (WFNS, Hunt and Hess, and Fisher); post-operative complications; vasospasm incidence; and Modified Rankin Scale (MRS) scores at discharge. Discrepancies were resolved by consensus. Patients who received treatment only for the ruptured aneurysm, with no intervention for the additional unruptured aneurysms, were analyzed separately and not included in the comparative analysis between the single-stage and multiple-stage groups.

Data analysis was performed using Python (3.11.4) with the following libraries utilized: Pandas (2.0.3), NumPy (1.24.3), and Matplotlib (3.7.1) for data manipulation and cleaning, numerical operations on data arrays, and for generating plots and figures to visualize the data distributions and analysis results. Specific analyses included descriptive statistics (mean, standard deviation, and range) for continuous variables such as age, length of operation, and hospital stay; frequency and percentage calculations for categorical data such as Hunt and Hess grades, Fisher grades, and WFNS grades; and outcomes measured by the MRS and detailed examination of aneurysm characteristics, including number, size, and location, as well as treatment modalities. Further analysis was conducted to compare the outcomes between patients undergoing single-stage and multiple-stage interventions for the treatment of MIAs. Continuous variables, such as the length of hospital stay, were analyzed using independent *t*-tests to assess differences in means between the two groups. Categorical variables were analyzed using chi-square tests to determine the association between the type of intervention and these outcomes. A *p*-value of less than 0.05 was considered statistically significant. Due to the retrospective nature and limited cohort size, no a prior sample size calculation was performed. This limitation is acknowledged in the discussion.

## 3. Results

### 3.1. Patient Demographics, Risk Factors, and Symptoms

**Figure 1:**
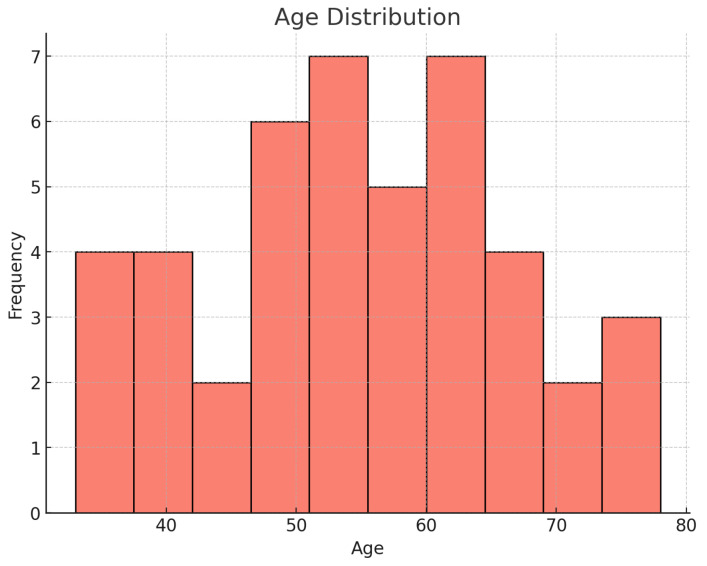
Distribution of ages.

**Figure 2:**
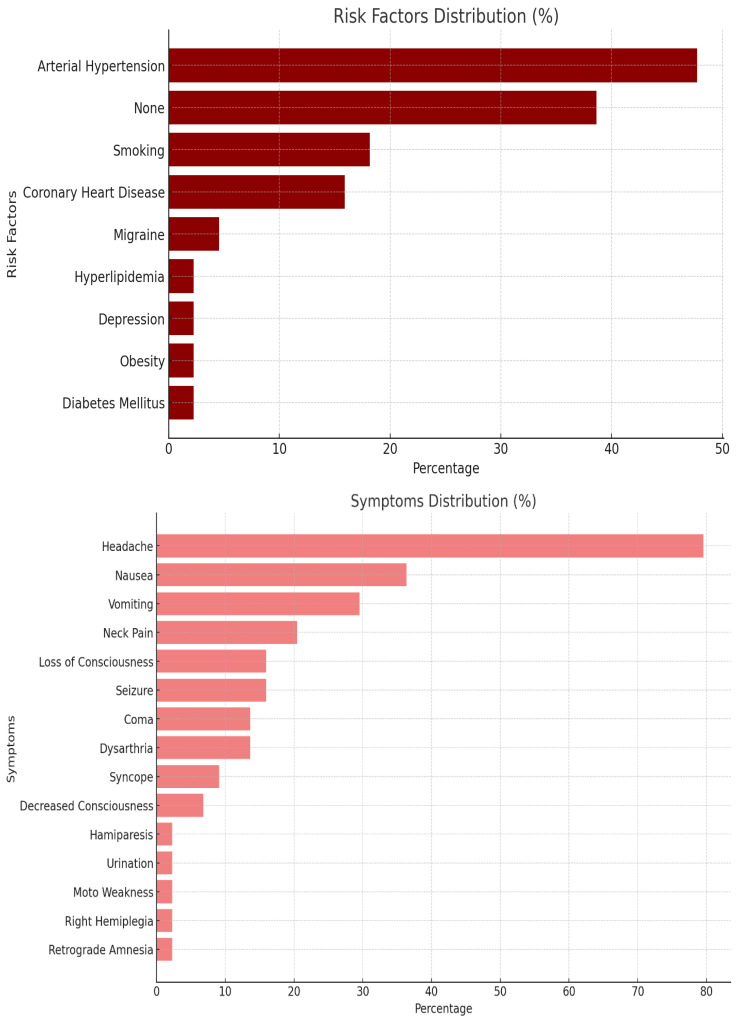
Risk factors and symptoms distribution.

In this study, we analyzed a cohort of 44 patients diagnosed with subarachnoid hemorrhage (SAH) and additional unruptured cerebral aneurysms. The mean age of the patients was 54.5 ± 12.0 years, ranging from 33 to 78 years, with the majority being female (68.18%) (Figure 1). Family history related to cerebral aneurysms was noted in 38.64% of the cases. Risk factor analysis revealed that the most prevalent was arterial hypertension (47.73%), while 38.64% had no risk factors, and smoking was reported in 18.18% of the cases. The symptoms most frequently observed were headaches, reported by 79.55% of patients, followed by nausea (36.36%) and vomiting (29.55%). Neck pain and seizure were noted in 20.45% and 15.91% of the patients, respectively (Figure 2) (Table 1).

### 3.2. Severity of SAH

**Figure 3:**
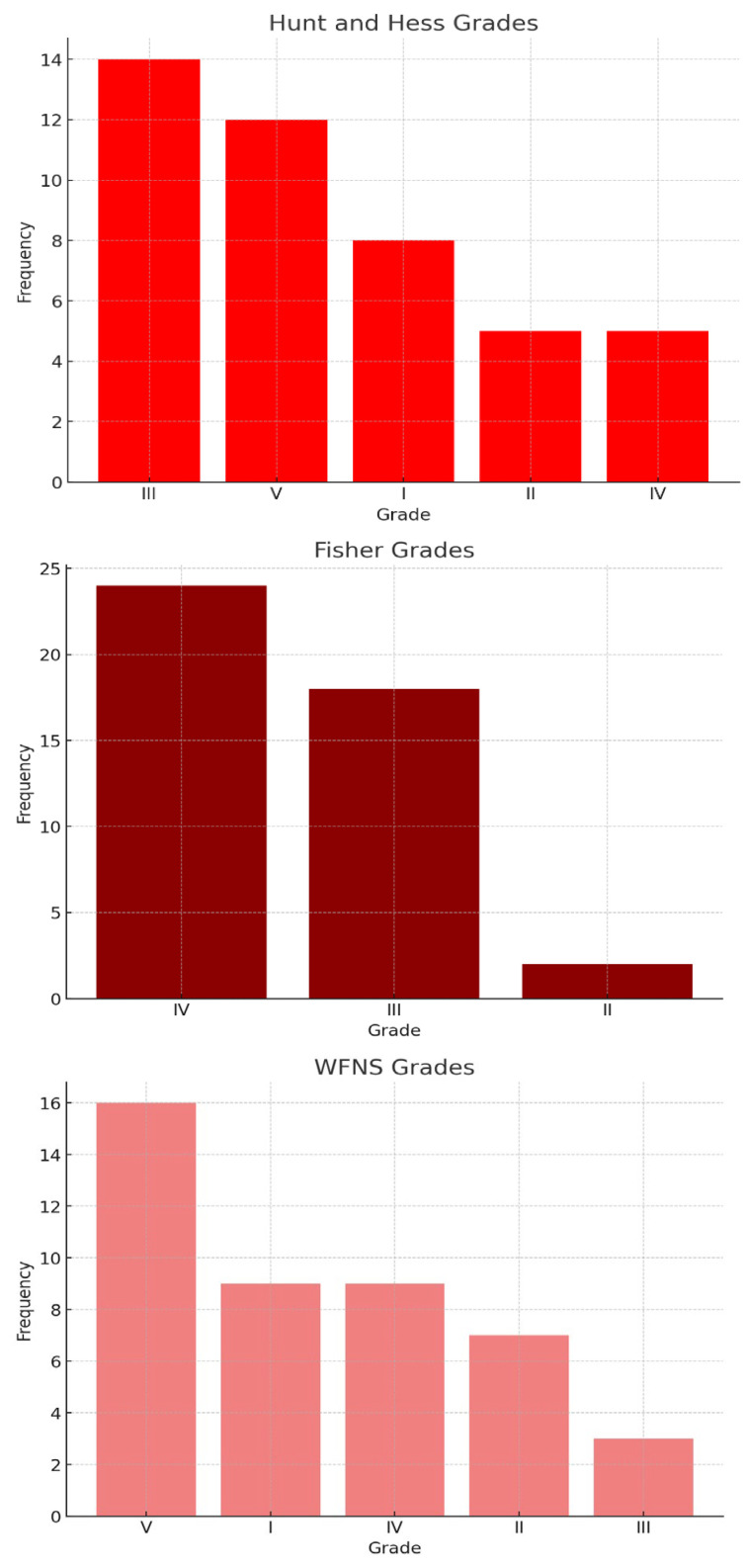
Hunt and Hess, Fisher, and WFNS grading scales.

The distribution of severity at presentation was quantified using the Hunt and Hess, Fisher, and WFNS grading scales. For the Hunt and Hess grades, 8 patients were assessed at Grade I, 5 at Grade II, 14 at Grade III, 5 at Grade IV, and 12 at Grade V, indicating a broad spectrum of initial severity. According to the Fisher scale, which predicts the risk of vasospasm based on initial CT findings, no patients were classified as Grade I, 2 were Grade II, 18 were Grade III, and the majority, 24 patients, were Grade IV. The WFNS grades, reflecting both the Glasgow Coma Scale score and the presence of motor deficits, showed 9 patients at Grade I, 7 at Grade II, 3 at Grade III, 9 at Grade IV, and 16 at Grade V, suggesting significant neurological impairment in many patients at admission (Figure 3).

### 3.3. Aneurysm Characteristics

**Figure 4:**
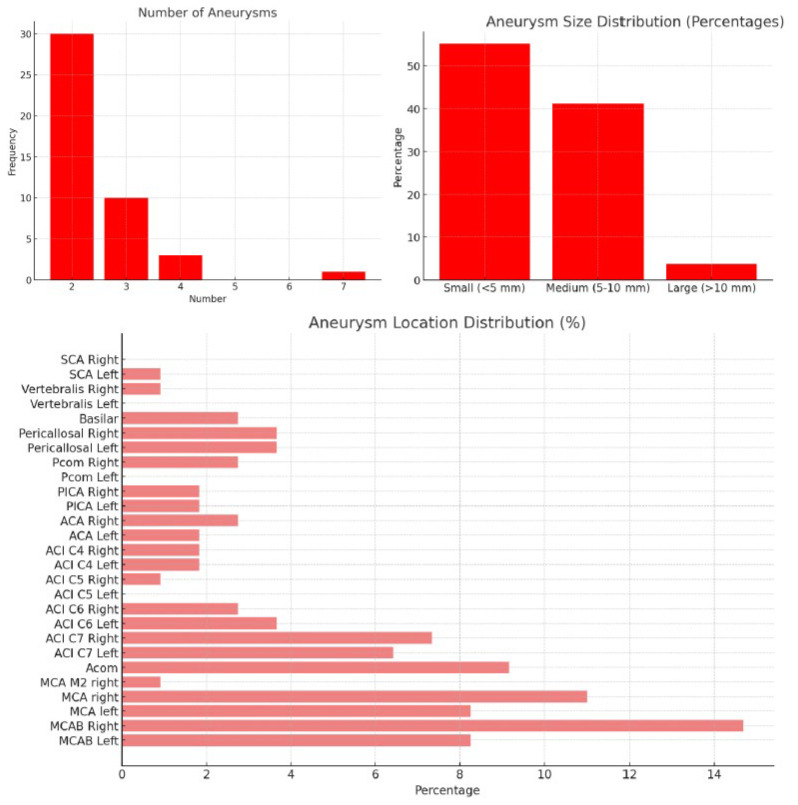
Number of aneurysms (**top left**), aneurysm size (**top right**), and aneurysm locations distribution.

In the analysis of aneurysm characteristics among our 44 patients, we observed a total of 109 aneurysms. The majority of patients (30) had two aneurysms, 10 patients had three aneurysms, 3 patients had four, and 1 patient had seven aneurysms (Figure 4). The categorization of aneurysm sizes based on their largest diameter showed that 59 aneurysms (55.14%) were classified as small (<5 mm), 44 (41.12%) as medium (5–10 mm), and 4 (3.74%) as large (>10 mm). The most commonly affected locations included the right MCAB, right MCA, and Acom. The assessment of associated radiological features revealed that intracerebral or intraventricular hemorrhage was present in 54.55% of cases, and hydrocephalus was noted in 47.73% of patients.

### 3.4. Treatment Stages, Interventions, and Clinical Outcomes

**Figure 5:**
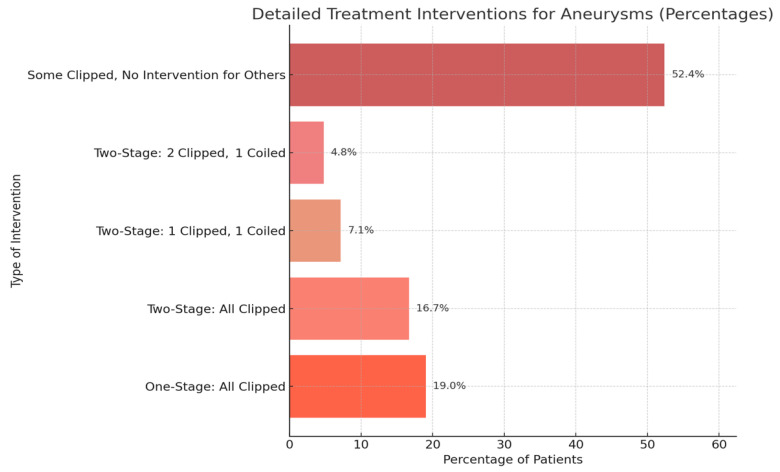
Treatment interventions and stages in SAH patients with multiple aneurysms.

Among the 44 patients, 23 (52.4%) underwent treatment of the ruptured aneurysm only and were not included in the comparative analysis of staged interventions. Of the remaining 21 patients, 8 received single-stage treatment of all aneurysms, and 13 received multiple-stage treatment, confirming true staged management of all aneurysms. Among the 109 aneurysms found in our cohort, 71 aneurysms were clipped, and 5 were treated using coiling. Specifically, eight (19.0%) patients underwent a one-stage clipping procedure for all detected aneurysms, while seven (16.7%) patients required a two-stage clipping intervention (Figure 5). Additionally, three (7.1%) patients had one of their two aneurysms clipped and the other coiled in a two-stage intervention. In a similar two-stage intervention, two (4.8%) patients had three aneurysms treated with a combination of clipping and coiling. Notably, 23 (52.4%) patients received clipping for the ruptured aneurysm only, with no intervention applied to the additional unruptured aneurysms.

**Figure 6:**
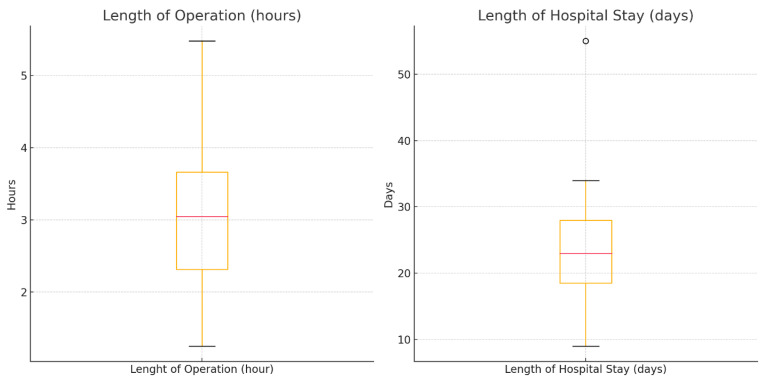
Length of operation and length of hospital stay of the whole cohort.

The mean length of operation was 3.09 h, with procedures ranging from 1.25 to 5.48 h (Table 2). Patients typically remained in the hospital for approximately 23.19 days, with durations extending from 9 to 55 days. Post-operative complications were observed in 77.27% of patients, while vasospasm was noted in 72.73% of cases, indicating high rates of these serious conditions. Neurological outcomes were varied, with the Modified Rankin Scale (MRS) scores distributed across all categories: 18.2% of patients had no symptoms (MRS 0), and 13.6% were deceased (MRS 6), illustrating the broad spectrum of outcomes (Figure 6).

**Figure 7:**
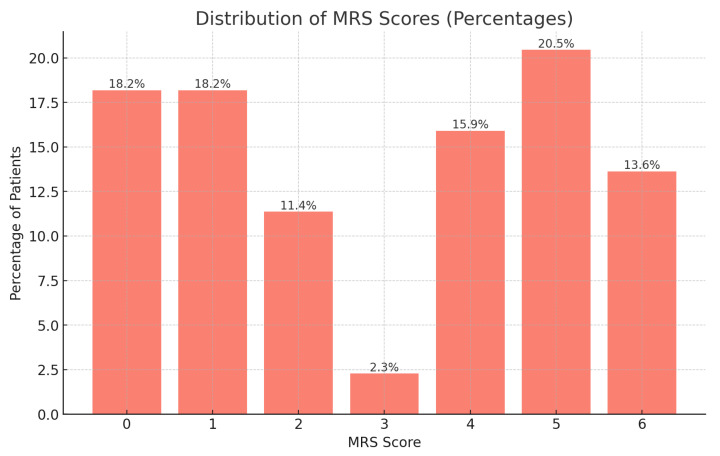
Distribution of MRS scores.

**Figure 8:**
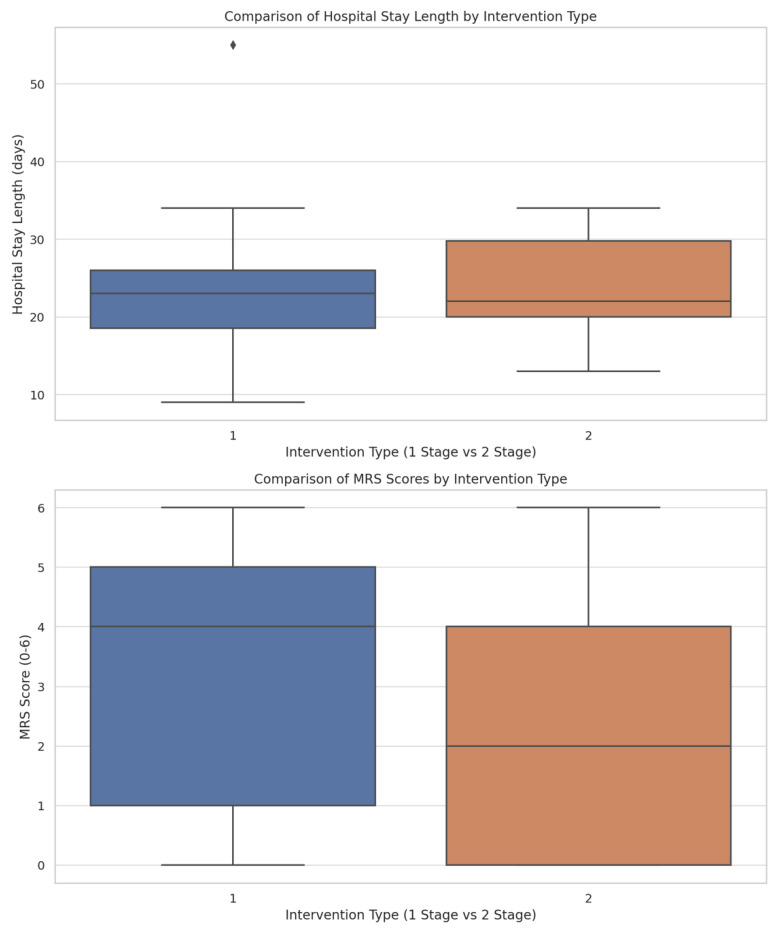
Boxplots illustrating the distribution of two key outcomes—length of hospital stay and MRS scores—between patients who underwent single-stage and multiple-stage interventions.

After analyzing the whole cohort for complications and MRS score, we categorized them based on whether they received single-stage or multiple-stage interventions. The length of hospital stay did not differ significantly between patients undergoing single-stage (mean = 22.8 ± 9.16 days) and multiple-stage interventions (mean = 22.6 ± 7.00 days, *p* = 0.646) (Figure 7). Vasospasm occurrence was notably higher in the single-stage intervention group (83.87%), while the multiple-stage intervention group had 46.15% occurrence, with a statistically significant difference (*p* = 0.028). MRS scores approached statistical significance but did not achieve it (*p* = 0.060), indicating a potential trend in outcome severity related to the type of surgical intervention. There was also a trend towards significance in the occurrence of neurological deficits post-operation between the two groups (*p* = 0.079) (Figure 8).

## 4. Discussion

### 4.1. Patient Demographics, Risk Factors, and Symptoms

The predominance of female patients in our cohort aligns with established epidemiological trends, particularly among postmenopausal women, suggesting a role of hormonal changes in compromising cerebrovascular integrity [19,20]. Positive family history in over one-third of the cases supports a genetic predisposition to aneurysm development, underscoring the potential value of targeted familial screening [21,22,23].

Clinically, headaches and hypertension were the most frequent presenting features. Hypertension, in particular, is a well-established risk factor for aneurysm rupture [24]. The notable prevalence of smoking further reinforces its role as a modifiable contributor to arterial damage and aneurysm pathogenesis [25].

### 4.2. Severity of SAH

The clinical grading in our cohort demonstrated considerable variability in the initial hemorrhage severity and associated complications. The predominance of higher Fisher grades (III and IV) reflects extensive hemorrhage and a heightened risk of vasospasm, a major post-SAH complication [26]. Similarly, a significant proportion of patients presented with severe Hunt and Hess and WFNS grades (III–V), underscoring the critical condition of many cases that require prompt, aggressive treatment [27]. The strong correlation between elevated WFNS grades and low Glasgow Coma Scale scores indicates severe neurological impairment at onset, complicating management and prognosis. These findings highlight the importance of intensive acute care and the potential need for improved early diagnostic and therapeutic approaches.

### 4.3. Aneurysm Characteristics

Our cohort predominantly exhibited small- to medium-sized aneurysms, consistent with epidemiological data showing that smaller aneurysms are more frequently detected, largely due to advances in high-resolution imaging [28,29]. The low incidence of large aneurysms (>10 mm) may reflect effective early detection and management or the natural rarity of larger aneurysms, which carry a higher rupture risk [30,31].

Aneurysms were most commonly located at the middle cerebral artery bifurcation (MCAB) and anterior communicating artery (Acom), sites known for hemodynamic stress and turbulent blood flow that are predisposed to aneurysm formation [32,33]. This underscores the importance of focused surveillance in these vascular regions, especially in patients with risk factors.

The frequent occurrence of associated radiological findings such as intracerebral hemorrhage and hydrocephalus highlights the severity of aneurysm rupture. Hydrocephalus, in particular, significantly affects prognosis and often requires interventions like ventricular drainage, which can impact clinical outcomes.

### 4.4. Treatment Stages, Interventions, and Clinical Outcomes

In managing patients with multiple intracranial aneurysms (MIAs), single-stage clipping is often preferred to minimize surgical exposure and recovery time [34,35]. However, two-stage interventions are utilized in more complex cases to safely address each aneurysm. The choice between clipping and coiling depends on the aneurysm characteristics, location, and patient-specific factors.

Nearly half of the unruptured aneurysms in our cohort were managed conservatively, reflecting the current neurosurgical debate weighing intervention risks against rupture potential [36].

Accurate identification of the ruptured aneurysm in MIAs is critical to effective treatment and reducing the rebleeding risk, a major determinant of morbidity and mortality. Hadjiathanasiou et al. developed a validated predictive score combining aneurysm-specific (size, location, and shape) and patient factors (age and hypertension) using a gradient boosting algorithm, which aids clinical decision-making when bleeding sources are unclear [13,15].

Endovascular single-stage treatment, as demonstrated by Andic et al., offers a technically feasible and efficient approach to managing multiple intracranial aneurysms in one session, achieving a high aneurysm obliteration success rate of 97.6% [11]. This approach benefits from the minimally invasive nature of endovascular techniques, reducing surgical trauma and potentially shortening recovery time. However, despite these advantages, Andic et al. also reported a significant complication rate, including a 15% mortality rate, underscoring the inherent risks in treating complex aneurysm presentations in a single procedure. The complications—ranging from thromboembolic events to hemorrhagic risks—highlight the necessity for meticulous patient selection and preoperative planning [11]. Particularly in anatomically challenging cases, such as bilateral internal carotid artery aneurysms, staged interventions may be safer. Staging allows for reduced procedural complexity per session, potentially lowering the risk of complications associated with prolonged operative times and extensive vascular manipulation [11].

From a surgical standpoint, Xing and Guo provided critical insights into tailoring intervention strategies based on aneurysm location relative to the Circle of Willis. Their study categorized patients into groups depending on whether aneurysms were unilateral, midline, or bilaterally distributed, influencing the decision between single-stage or multiple-stage surgeries. They found that patients with aneurysms confined to one side or along the midline typically had favorable outcomes following single-stage surgery, benefiting from reduced anesthesia exposure and faster recovery [14]. Conversely, patients with bilaterally located or anatomically complex aneurysms often required staged surgical interventions. This staged approach aims to minimize the cumulative surgical trauma and reduce risks such as cerebral vasospasm, prolonged brain retraction, and operative blood loss. Their findings emphasize that anatomical complexity and aneurysm distribution are pivotal in surgical planning, directly impacting patient outcomes and complication rates. Therefore, a flexible and individualized approach based on a detailed anatomical assessment is essential to optimize safety and efficacy in aneurysm management [14]. The continued relevance of microsurgical clipping, even in the endovascular era, is supported by recent findings, which reported the successful clipping of ruptured basilar artery perforator aneurysms [37]. Although our study focuses primarily on anterior circulation aneurysms, the principle of strategic selection based on anatomical feasibility is shared. Recent studies have shown favorable outcomes with coiling in small ruptured aneurysms, particularly in the anterior circulation [38,39].

While five aneurysms in our cohort were treated with coiling, the overwhelming preference for clipping reflects institutional expertise and local protocols during the study period. Some patients were considered for endovascular treatment but were deferred to surgical clipping based on aneurysm morphology, accessibility, or medical contraindications.

Single-stage interventions generally offer advantages like reduced patient risk exposure and resource utilization, especially when aneurysms are accessible via unilateral surgery or minimally invasive endovascular methods [11]. Hong et al. confirmed feasibility even for bilaterally located aneurysms accessible from a single craniotomy [16,17]. Nonetheless, the decision depends heavily on aneurysm complexity, location, and patient health, with deep or critical area aneurysms often necessitating staged approaches to avoid complications such as vasospasm or brain injury [15].

Although a higher incidence of vasospasm was observed in the single-stage group—possibly due to concentrated vascular manipulation and prolonged surgical stress, which can trigger endothelial disruption, inflammation, and altered cerebral blood flow—this finding may partially reflect the higher initial clinical severity in this group. Additionally, since the MRS score difference only approached statistical significance (*p* = 0.060), we urge caution in interpreting this trend as definitively clinically meaningful.

Asiltürk et al. advocated microsurgical clipping of all accessible aneurysms in a single session when anatomically feasible, a strategy aligned with our findings of comparable hospital stays between single- and multiple-stage surgeries [12]. However, this approach does not fully address the vasospasm risk. Conversely, Zhang et al. suggested that staged endovascular treatment may lower hemorrhagic and ischemic complication rates [18].

## 5. Limitations

While this study provides valuable insights, several limitations must be acknowledged. The retrospective design inherently limits the control over potential confounding factors and raises the risk of selection bias. Due to the limited sample size, we were unable to perform multivariable logistic regression to adjust for potential confounders, such as baseline WFNS or Hunt and Hess grades. This is a major limitation, and future larger studies are needed to isolate the impact of intervention type from the underlying clinical severity. Additionally, the absence of long-term follow-up data limits our ability to assess the sustained efficacy and durability of treatment modalities such as clipping and coiling, as well as long-term functional outcomes and complication rates. Future research should prioritize prospective, longitudinal studies with larger and more heterogeneous cohorts to address these gaps. Moreover, incorporating multi-center data would improve the generalizability and external validity of the findings by capturing variations in clinical practice and patient demographics across different healthcare settings.

## 6. Conclusions

This study offers valuable insights into the characteristics, treatment strategies, and outcomes of SAH patients with multiple intracranial aneurysms. Our analysis demonstrates that the choice between single-stage and multiple-stage interventions depends primarily on aneurysm complexity and patient condition. While multiple-stage approaches may reduce the risk of complications such as vasospasm and allow the tailored management of each aneurysm, single-stage interventions remain essential for certain complex cases. Ultimately, treatment decisions balance the risk of intervention against the natural history of the aneurysm, underscoring the need for individualized, case-specific strategies to optimize patient outcomes.

## Figures and Tables

**Table 1:** (**a**) Patient demographics, family history, risk factors, and symptoms. (**b**) Aneurysm characteristics and radiological features.

**a**			
**Category**	**Metric**	**Count**	**Percentage**
**Patient Demographics**	Female	35	68.18%
	Male	9	20.45%
	Age (mean)	54.5 ± 12.0	
	Age (Range)	33–78	
**Family History**	Yes	17	38.64%
	No	19	43.18%
	Unknown	8	18.18%
**Risk Factors**	Arterial Hypertension	21	47.73%
	None	17	38.64%
	Smoking	8	18.18%
	Coronary Heart Disease	7	15.91%
	Migraine	2	4.55%
	Depression	1	2.27%
	Diabetes Mellitus	1	2.27%
	Hyperlipidemia	1	2.27%
	Obesity	1	2.27%
**Symotoms**	Headache	35	79.55%
	Nausea	16	36.36%
	Vomiting	13	29.55%
	Neck Pain	9	20.45%
	Seizure	7	15.91%
	Loss of Consciousness	7	15.91%
	Coma	6	13.64%
	Dysarthria	6	13.64%
	Decreased Consciousness	3	6.82%
	Syncope	4	9.09%
	Hemiparesis	1	2.27%
	Motor Weakness	1	2.27%
	Retrograde Amnesia	1	2.27%
	Right Hemiplegia	1	2.27%
	Urination	1	2.27%
**b**			
**Aneurysm Sizes**	- Small (<5 mm)	59	55.14%
	- Medium (5–10 mm)	44	41.12%
	- Large (>10 mm)	4	3.74%
**Top Aneurysm Locations**	MCAB Right	16	14.95%
	MCA Right	12	11.21%
	Acom	10	9.35%
**Radiological Features**	Intracerebral Hemorrhage	24	54.55%
	Hydrocephalus	21	47.73%

**Table 2:** Outcome measures.

Outcome Measures				
**Measure**	**Mean**	**SD**	**Min**	**Max**
Length of Operation (hours)	3.09	1.01	1.25	5.48
Length of Hospital Stay (days)	23.19	8.56	9.00	55.00
**Measure**	**Yes**		**No**	
Post-op Complications (%)	77.27%		22.73%	
Vasospasm (%)	72.73%		27.27%	

## Data Availability

The data presented in the following study are available from the corresponding authors upon request.
